# Evaluation of Monomer-containing Isocyanate Groups on Stabilizing Demineralized Dentin Matrix Against Bond Interface Degradation

**DOI:** 10.3290/j.jad.b5546415

**Published:** 2024-07-05

**Authors:** Zhenyu Yang, Jing Gao, Kai Tang, Longyan Duan, Shiqi Dai, An Chen, Wei Zhou, Jihua Chen

**Affiliations:** a Dentist, Department of Stomatology, 923th Hospital of the Joint Logistics Support Force of PLA, Nanning 530021, China; State Key Laboratory of Military Stomatology & National Clinical Research Center for Oral Diseases & Shaanxi Key Laboratory of Stomatology, Department of Prosthodontics, School of Stomatology, The Fourth Military Medical University, Xi’an, 710032, China. Performed the experiment, co-wrote and proofread the manuscript, with the 2nd author contributed equally to this work.; b Dentist, State Key Laboratory of Military Stomatology & National Clinical Research Center for Oral Diseases & Shaanxi Key Laboratory of Stomatology, Department of VIP Dental Care, School of Stomatology, The Fourth Military Medical University, Xi’an, 710032, China. Performed the experiment, co-wrote and proofread the manuscript, and with the first author contributed equally to this work.; c MSc Student, State Key Laboratory of Military Stomatology & National Clinical Research Center for Oral Diseases & Shaanxi Key Laboratory of Stomatology, Department of Prosthodontics, School of Stomatology, The Fourth Military Medical University, Xi’an, 710032, China. Co-wrote and proofread the manuscript.; d Research Fellow, State Key Laboratory of Military Stomatology & National Clinical Research Center for Oral Diseases & Shaanxi Key Laboratory of Stomatology, Department of Prosthodontics, School of Stomatology, The Fourth Military Medical University, Xi’an, 710032, China. Co-wrote and proofread the manuscript.; e PhD Student, State Key Laboratory of Military Stomatology & National Clinical Research Center for Oral Diseases & Shaanxi Key Laboratory of Stomatology, Department of Prosthodontics, School of Stomatology, The Fourth Military Medical University, Xi’an, 710032, China. Co-wrote and proofread the manuscript.; f Dentist, Department of Stomatology, 923th Hospital of the Joint Logistics Support Force of PLA, Nanning 530021, China. Proofread and revised the manuscript.; g Dentist, State Key Laboratory of Military Stomatology & National Clinical Research Center for Oral Diseases & Shaanxi Key Laboratory of Stomatology, Department of Prosthodontics, School of Stomatology, The Fourth Military Medical University, Xi’an, 710032, China. Co-wrote and proofread the manuscript.; h Professor, State Key Laboratory of Military Stomatology & National Clinical Research Center for Oral Diseases & Shaanxi Key Laboratory of Stomatology, Department of Prosthodontics, School of Stomatology, The Fourth Military Medical University, Xi’an, 710032, China. Co-wrote and proofread the manuscript.; # Equal contributors

**Keywords:** isocyanate, dentin, collagen, proteolysis, hydroxyproline

## Abstract

**Purpose:**

To evaluate the effect of urethane methacrylate precursor (UMP) on the enzymatic resistance of demineralized dentin (DD) matrices.

**Materials and Methods:**

Experimental treatments containing 0 (control), 1, and 5 mmol/L UMP dissolved in an acetone (Ace) solution were formulated. Dentin matrix specimens were demineralized in vitro and immersed in the experimental treatments for 1 h. The treated specimens were then stored in 0.1 mg/mL collagenase solution for 24 h, after which their dry mass loss and hydroxyproline (HYP) release were assessed. The swelling ratios of specimens in each group were also evaluated. The interaction between UMP and the dentin matrix was observed using field-emission scanning electron microscopy (FE-SEM). Endogenous enzyme activity in dentin was evaluated using confocal laser scanning microscopy (CLSM).

**Results:**

Compared with the other treatment groups, treatment with 1 mM and 5 mM UMP-Ace significantly decreased the dry mass loss, HYP release and swelling ratio of the DD matrix (p < 0.05). FE-SEM and CLSM observations showed that treatment with UMP-Ace protected the structure of the dentin matrix and decreased porosity within the dentin-collagen network.

**Conclusion:**

Treatment with 1 mM and 5 mM UMP-Ace protects DD matrix against collagenase degradation and may be clinically useful for improving the durability of the hybrid layer.

Compared with enamel, natural dentin has a heterogeneous composition and structure; thus, its bonding mechanism is more complex.^15^ Current bonding systems can achieve sufficient immediate dentin bond strength, but the literature shows that the durability of these bonding systems is not ideal. Although different dentin bonding systems have different compositions and formulas, adhesive bonding to dentin is mainly achieved through the hybrid layer. After dentin is demineralized by phosphoric acid or other organic acidic molecules, the minerals in the superficial area of the dentin dissolve, forming a loose and porous three-dimensional network structure. The resin monomer penetrates into the voids generated by the dissolution of minerals and hybridizes with collagen.^6^ When the adhesive is cured, a hybrid layer structure is formed to provide micromechanical interlocking.^22,33^

However, the esterase hydrolysis of resin-based adhesives^20^ and endogenous enzymatic hydrolysis of collagen fibers occur in the hybrid layer, effectively rendering it the weakest link in dentin bonding. The main sources of water for esterase hydrolysis are dentinal tubule fluid, water absorbed by hydrophilic monomers in the adhesive, and water introduced by wet-bonding technology.^24,25^ Water triggers the hydrolysis of the resin adhesive by esterase, resulting in the destruction of the sealing of the bonding interface, which affects the bonding durability. The endogenous enzymatic hydrolysis of collagen fibers in the hybrid layer is mainly related to matrix metalloproteinases (MMPs) and cysteine cathepsins (CTs). Although MMPs and CTs coexist in dentin, the former has increased, indicating a more relevant role in dentin matrix degradation.^26^ During dentin development and mineralization, MMPs are secreted by odontoblasts into the dentin matrix in the form of zymogens, and retained in the mineralized mature dentin.^4,17,29,30^ Regardless whether an etch-and-rinse (E&R) system or self-etch system is used, some exposed collagen fibers unwrapped by the adhesive in the hybrid layer;^16^ thus, these fibers are exposed to MMPs activated by the etchant or acidic monomers.^9^

To prevent the hydrolysis of the hybrid layer and promote bonding stability, scholars have conducted extensive research, mainly focusing on three aspects: reducing or removing residual water in the hybrid layer, inhibiting the activity of MMPs, and forming collagen crosslinks. Natural polyphenolic compounds such as proanthocyanidins, genipin and green tea extract (epigallocatechin gallate, EGCG) have been shown to inhibit MMPs and CTs,^1,2,34,36^ but the inhibitory mechanism remains incompletely understood. Epasinghe et al^28^ showed that proanthocyanidins have a stronger ability than chlorhexidine to inhibit proteolytic enzyme activity. In efforts to increase collagen crosslinking, Bedran-Russo and Tezvergil-Mutluay found that carbodiimide (EDC), which contains RN=C=NR functional groups, can form stable covalent amide bonds between collagens, thereby improving the mechanical properties of the dentin matrix, however, the clinical operation time was too long.^3,31^

Chemical cross-linking can introduce covalent or non-covalent bonds between molecules to increase the degree of cross-linking between collagen molecules, thereby improving the mechanical properties, enzymatic hydrolysis resistance and heat resistance of collagen.^21,32^ We previously synthesized a –NCO-terminated urethane methacrylate precursor (UMP) with a UMP-based adhesive that ultimately demonstrated promising dentin bond strength.^35^ The reaction between the –NCO groups of the UMP and the –NH2 groups of collagen, introduced curable double bonds within the collagen fibrils that could be used to prepare hybrid collagen polymer composites via a copolymerization approach.^13^ However, whether chemical cross-linking affects enzyme activity is unknown. Thus, this study aimed to evaluate the effect of UMP on stabilizing the DD matrix against degradation.

## Materials and Methods

### Preparation of the UMP-acetone (Ace) Treatments

**Fig 1:**
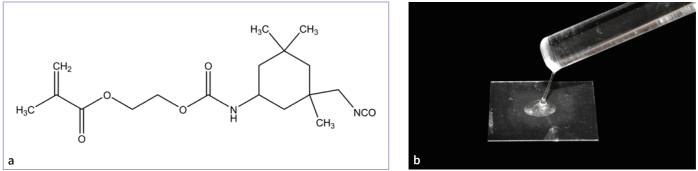
Chemical structural formula and gross appearance of UMP. (a) The chemical structure of UMP. (b) Gross appearance of UMP monomer.

As shown in Figure 1, UMP was synthesized via the reaction of isophorone diisocyanate (IPDI) and HEMA in the presence of a catalyst.^37^ The reaction system was cooled to room temperature, mixed with silica gel, and separated by column chromatography (petroleum ether/ethyl acetate = 5/1, v/v). Rotary evaporation of the solvent was performed to obtain a colorless oil. Because the UMP synthesis and purification method has been introduced in the previous paper of our research group,^37^ it is not described in detail in this manuscript. Different concentrations of UMP were added to Ace, and thoroughly mixed by ultrasonic vibration to prepare the following experimental treatments:

- Group 1: Ace without UMP (control group);
- Group 2: 1 mmol/L UMP-Ace solution (1 mM UMP); and
- Group 3: 5 mmol/L UMP-Ace solution (5 mM UMP).

### Sample Preparation

The study protocol (IRB-REV-2018011) was approved by the Forth Military Medical University Ethics Committee. Freshly extracted third molars were obtained from the Department of Oral Surgery. Fifty intact and healthy isolated teeth were selected, and the crown enamel was fully removed with a diamond cutting machine in the direction perpendicular to the long axis of the tooth 1 mm below the enamel-dentin junction to expose the dentin. Twenty isolated teeth were cut into 36 dentin beams (1 mm × 1 mm × 10 mm) along the long axis of the tooth, and the remaining 30 teeth were cut into 48 dentin slabs (6 mm × 6 mm × 1 mm) perpendicular to the long axis of the tooth.

### Loss of Dry Mass and Release of Hydroxyproline (HYP)

The dentin beams were immersed in 10% phosphoric acid solution for 24 h to achieve complete demineralization. The collagen beams were randomly divided into three groups (n = 6), and soaked in the three UMP-Ace experimental treatments for 5 min. The beams were then dehydrated in a vacuum drying oven for 24 h to a constant weight, and the weight of each sample was recorded (W0). Thereafter, the dentin samples were completely soaked in deionized water to restore their original shape, placed into an Eppendorf tube filled with 1.5 mL of 0.1 mg/mL collagenase solution (LIFE Invitrogen, USA), and maintained at 37° for 24 h. After enzymatic hydrolysis, 500 µL of the supernatant was collected, and the HYP content in the supernatant was detected at a wavelength of 550 nm on a microplate reader with an HYP detection kit (Solarbio, Beijing, China) according to the manufacturer’s instructions. The dentin beams at the bottom of the Eppendorf tubes were collected, dried and reweighed individually (W1). The percentage dry mass loss was calculated as: (W1 – W0) / W0 × 100%.

### Swelling Ratio

The DD beams were treated with the three UMP-Ace experimental treatments for 5 min and then allowed to swell in PBS (pH= 7.4) for 24 h. The excess water on the surface of the specimens was blotted dry with a filter paper, and the specimens were quickly weighed to determine the weight of the swollen sample. Immediately afterwards, the dentin beams were incubated in a large amount of distilled water for 20 min to remove the buffer salts. Each group of samples was subsequently recovered ventilated, and dried to a constant weight, which was recorded as the weight of the dry sample. The swelling ratio was calculated as the ratio of the weight of the swollen sample to that of the dry sample.

### Morphological Observation of Demineralized Dentin

Thirty dentin slabs were obtained and divided into three experimental groups (n = 10). The middle of the bottom side of each slab was notched for subsequent fracturing. The polished dentin surfaces were etched for 15 s with 37% phosphoric acid gel (Scotchbond Universal Etchant, 3M ESPE, St. Paul, MN, USA), rinsed with water for 30 s, treated for 60 s, and then rinsed according to the experimental conditions described above. Dentin slabs in each group were evaluated with (n = 5) or without (n = 5) 0.1 mg/mL collagenase (Type IV, 160 units/mg, Aladdin, Shanghai, China) digestion. The slabs were fractured, mounted on aluminum stubs, and coated with carbon. The fractured cross-sections were examined under a field-emission environmental scanning electron microscope at 2000× magnification in secondary-electron (SE) or backscattered-electron (BSE) mode.

### Zymography of Demineralized Dentin

Dentin in situ enzyme activity was detected using a DQ gelatin kit (Invitrogen Corporation, EvoQuest TM Laboratory Services, USA). A fluorescent collagen working solution was prepared with 1 mg/mL DQ gelatin solution, anti-fluorescence quenching agent H-1200, and 1× buffer at a ratio of 1:1:8. The prepared dentin slabs were randomly divided into four groups as follows: non-demineralized dentin, DD, 1 mM UMP-Ace-pretreated DD, and 5 mM UMP-Ace-pretreated DD. Demineralization was achieved by etching with 37% phosphoric acid gel for 15 s. Specimens in the non-demineralized and DD groups were soaked in deionized water, whereas those in the other groups were soaked in different concentrations of the UMP-Ace solutions for 5 min. Thereafter, the specimens were rinsed with running water for 1 min, and ultrasonically vibrated for 10 min to remove residues on the dentin surface. Twenty microliters of the DQ gelatin working solution were evenly added to the surface of each specimen, which was subsequently covered with a cover glass and incubated for 48 h at 37°C in a dark room with 100% humidity. The green fluorescence intensity of the dentin surface was then detected. The absorption wavelength was 488 nm, the scanning interval was 0.5 μm, and the scanning depth was 20 μm. The intensity of green fluorescence on the dentin surface was calculated using Image J software, and three discrete 50 × 50 μm^2^ areas were selected from each specimen to calculate the fluorescence percentage. The average value of the three calculations was taken as the result of one experiment, and each experiment was repeated six times. Collagenase activity was expressed as the percentage of the fluorescence area on the dentin interface.

### Statistical Analysis

Statistical analyses were performed using SPSS 22.0. Data for dry mass, release of HYP, swelling ratio, and percentage of fluorescence area were expressed as means ± standard deviations. The Shapiro–Wilk test and the Bartlett test were performed sequentially to ascertain the normality and the homoscedasticity assumptions of the respective data sets. One-way analysis of variance was used to compare the loss of dry mass, release of HYP, swelling ratio, and percentage of fluorescence area, followed by the least-significant difference (LSD), and Tamhane. Two-sided tests were conducted with α = 0.05.

## Results

The dry mass loss and HYP release of the specimens are shown in Table 1. Dentin slabs pretreated with 5 mM UMP-Ace showed the lowest percentage loss of dry mass (18.30 ± 0.39%), followed by the 1 mM UMP-Ace-treated (27.74 ± 2.10%) and control (62.15 ± 3.77%) groups (p < 0.05). Similar to the trend of dry mass loss, the 5 mM UMP-Ace -treated dentin beams liberated the lowest amount of HYP (5.24 ± 0.26 μg HYP/mg dentin), and the control group (8.70 ± 0.78 μg HYP/mg dentin) retained a significantly higher amount of HYP than the 1 mM UMP-Ace-treated group (6.06 ± 0.47) (p < 0.05).

**Table 1:** Dry mass loss and hydroxyproline release from demineralized dentin beams

	Dry mass loss (%)	HYP release (μg/mg)
Control 1 mM 5 mM	62.15 ± 3.77 ^A^ 27.74 ± 2.10 ^B^ 18.30 ± 0.99 ^C^	8.70 ± 0.78 ^A^ 6.06 ± 0.47 ^B^ 5.24 ± 0.26 ^C^

Mean percent loss of dry mass and hydroxyproline release from demineralized dentin beams treated with different UMP-Ace solutions after 24 h of collagenase degradation. Differences between groups marked with the same letters are not statistically significant (p >0.05).

**Fig 2:**
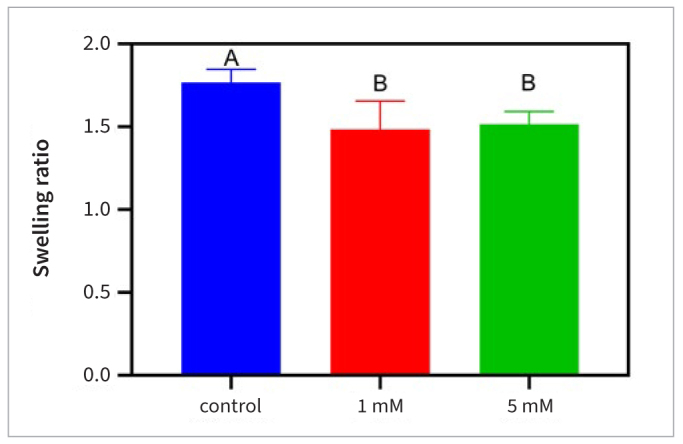
Swelling ratio of demineralized dentin beams treated with different UMP-Ace solutions. Differences between groups marked with the same letters are not statistically significant (p > 0.05).

The swelling ratio results are shown in Figure 2. The DD beams treated with 1 and 5 mM UMP-Ace showed significantly lower weight gains when swollen compared with the control groups (p < 0.05).

**Fig 3:**
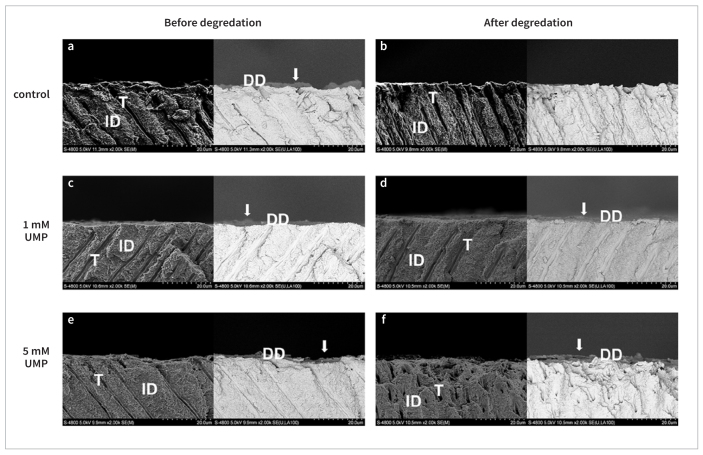
Representative photomicrographs (2000×) of demineralized dentin (DD) slabs from all groups before (Fig. 3a, 3c, 3e) and after (Fig. 3b, 3d, 3f) collagenase digestion obtained using SEM in secondary-electron (SE, left) and backscattered-electron (BSE, right) modes. Compared with the control, all UMP-treated groups showed intact DD after collagenase degradation regardless of the rinse applied. DD: demineralized dentin; ID: intact dentin; T: dentin tubules.

Before collagenase digestion, all groups showed DD layers with a similar morphology. Specifically, collagen fibrils were observed under SE mode, and a dark layer was noted under BSE mode (Fig 3). After digestion, DD samples treated with UMP-Ace remained intact, regardless of the treatment concentration.

**Fig 4:**
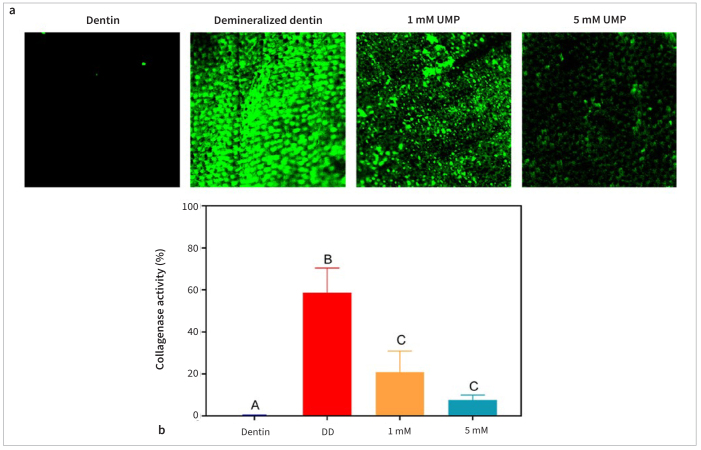
Zymography of demineralized dentin (DD) and collagenase inhibition. (a) Representative images (100×) derived from the zymography of DD after different treatment. (b) Quantification of collagenase activity. Differences between columns marked with the same letters are not statistically significant (p > 0.05).

The zymography results of the dentin obtained after different treatments (Fig 4) revealed that natural mineralized dentin showed almost no detectable green fluorescence. By contrast, DD exhibited high fluorescence intensity owing to the exposure of apatite-entrapped endogenous MMPs in the natural dentin and their activation after phosphoric acid etching. However, enzyme activity decreased significantly after treatment with UMP-Ace (p < 0.05).

## Discussion

Demineralized dentin collagen is mainly composed of type I collagen, which has a structure that is rich in active amino (-NH2) groups.^27^ These active groups can be used as targets to design adhesive functional monomers that can form strong covalent bonds to improve the dentin adhesive effect. Biomaterials containing isocyanate groups (-NCO) are widely used in the field of biomedicine because of their ability to covalently bond with -NH2 groups in collagen.^5^ Natural dentin contains a variety of endogenous enzymes, such as MMP-2, -3, -8, -9, etc.^23^ Endogenous MMPs are released during the bonding process after dentin demineralization, which destroys the bonding effect.^14^ In this study, we aim to mitigate this enzymatic effect by chemical bonding.

Natural mineralized dentin contains many apatite-embedded endogenous enzymes, as well as MMPs.^6^ These collagenolytic enzymes are activated after etching with mild acids, acidic functional monomers, or acidic substances derived from bacterial metabolism.^11^ Compared with the control matrix, the UMP-Ace-treated dentin-collagen matrices showed significantly less HYP release after exposure to bacterial collagenase, which is indicative of improved enzymatic stability and may be attributed to several factors. Collagen unwinding is necessary to expose unraveled fibrils to the catalytic domains of some MMPs, and we believe that UMP-Ace treatment inhibits this unwinding process.^8,12,32^

Compared with that of the control group, the swelling ratio of the two UMP-Ace treatment groups was significantly reduced, indicating that the porosity of the collagen network was reduced and that the penetration of water and buffer salts into the dentin matrix was hindered. A low swelling ratio may reduce the uptake of exogenous enzymes, thereby enhancing the resistance of the collagen in the dentin matrix to enzymatic hydrolysis.^10^

To further test collagen crosslinking and biostability in a clinically relevant situation, we etched dentin slabs with a commercial phosphoric acid gel for 15 s to create DD layers, which were subsequently treated according to the experimental procedures described above. The ability of UMP to penetrate the DD layers and induce collagen crosslinking over a clinically relevant period (60 s) was evaluated by SEM. In situ observations of the DD layers showed evidence of enhanced protection by UMP, which is consistent with the above quantitative biostability results.

MMPs are calcium- and zinc-dependent endopeptidases that are present in dentin as zymogens or proenzymes.^19^ In the E&R system, some MMPs are denatured or inactivated during 37% phosphoric acid etching (pH = 0.7); this effect is only temporary, however, and the MMPs are eventually reactivated under the mild acidity of the subsequent E&R adhesive.^18^ At this point, the bottom of the hybrid layer is not wrapped by the resin monomer, and the exposed collagen fibers are easily hydrolyzed by MMPs; this hydrolysis destroys the integrity of the hybrid layer and affects the bonding durability of dentin.^7^ Therefore, the inhibitory effect of UMP on MMPs was further verified by in situ zymography. The results showed weaker green fluorescence in the UMP-treated dentin groups compared with that in the control group, indicating lower MMP activity in these areas. This result demonstrates that UMP inhibits MMPs and downregulates their gelatinolytic activity.

## Conclusion

The results of the present study suggest that treatment with 1 mM and 5 mM UMP-Ace solution can protect DD matrices against collagenase degradation. This also provided theoretical support for the improvement of the enzymatic degradation resistance of resin-dentin restorations and laid a foundation for the application of UMP in dental bonding. Further research is required to elucidate the corresponding mechanism.

## Clinical Relevance

The enzymatic resistance of UMP means it slows down the degradation of the hybrid layer. Translation of this in vitro proof-of-concept strategy into a clinically applicable technique in vivo may improve the durability of dentin bonding and prolong the service life of the restoration.
